# The preprogrammed anti-inflammatory phenotypes of CD11c^high^ macrophages by *Streptococcus pneumoniae* aminopeptidase N safeguard from allergic asthma

**DOI:** 10.1186/s12967-023-04768-2

**Published:** 2023-12-11

**Authors:** Shifei Yao, Danlin Weng, Yan Wang, Yanyu Zhang, Qi Huang, Kaifeng Wu, Honghui Li, Xuemei Zhang, Yibing Yin, Wenchun Xu

**Affiliations:** 1 Key Laboratory of Laboratory Medical Diagnostics Designated by the Ministry of Education, School of Laboratory Medicine, Chongqing Medical University, Chongqing, 400016 China; 2 Department of Laboratory Medicine, The First People’s Hospital of Zunyi City (The Third Affiliated Hospital of Zunyi Medical University), Zunyi, 563000 China

**Keywords:** Hygiene hypothesis, Allergic asthma, *Streptococcus pneumoniae* aminopeptidase N, Macrophage, Oxidation phosphorylation

## Abstract

**Background:**

Early microbial exposure is associate with protective allergic asthma. We have previously demonstrated that *Streptococcus pneumoniae* aminopeptidase N (PepN), one of the pneumococcal components, inhibits ovalbumin (OVA) -induced airway inflammation in murine models of allergic asthma, but the underlying mechanism was incompletely determined.

**Methods:**

BALB/c mice were pretreated with the PepN protein and exposed intranasally to HDM allergen. The anti-inflammatory mechanisms were investigated using depletion and adoptive transfer experiments as well as transcriptome analysis and isolated lung CD11c^high^ macrophages.

**Results:**

We found pretreatment of mice with PepN promoted the proliferation of lung-resident F4/80^+^CD11c^high^ macrophages in situ but also mobilized bone marrow monocytes to infiltrate lung tissue that were then transformed into CD11^high^ macrophages. PepN pre-programmed the macrophages during maturation to an anti-inflammatory phenotype by shaping the metabolic preference for oxidative phosphorylation (OXPHOS) and also inhibited the inflammatory response of macrophages by activating AMP-activated protein kinase. Furthermore, PepN treated macrophages also exhibited high-level costimulatory signaling molecules which directed the differentiation into Treg.

**Conclusion:**

Our results demonstrated that the expansion of CD11c^high^ macrophages in lungs and the OXPHOS metabolic bias of macrophages are associated with reduced allergic airway inflammation after PepN exposure, which paves the way for its application in preventing allergic asthma.

**Supplementary Information:**

The online version contains supplementary material available at 10.1186/s12967-023-04768-2.

## Background

Asthma is a complex disease with a marked heterogeneity that globally affects more than 350 million people, and its prevalence has dramatically increased over the past few decades [1]. The hygiene hypothesis states that environmental alterations and lack of early life stages microbial exposure are responsible for this increased incidence [2, 3]. Therefore, altering microbial exposure to limit or prevent asthma is one approach within the scope of clinical treatments.

For example, infection with Murid herpesvirus 4 (MuHV-4), *Lactobacillus rhamnosus* GG or *S. pneumoniae* can inhibit the development of experimental allergic asthma [4–6]. However, the use of live bacteria or viruses may result in undesirable side effects such as overt infection and are counter-productive. In lieu of this, a standardized, low-endotoxin alkaline lysate (OM-85) of 21 bacterial strains from 5 genera (*Moraxella, Hemophilus, Klebsiella, Staphylococcus* and *Streptococcus*) has shown efficacy in preventing respiratory tract infections and can protect against experimental allergic asthma [7]. However, the composition of OM-85 is complex, and there is still an insufficient number of well-conducted clinical trials confirming its safety and efficacy in asthma prevention and treatment [8, 9]. Studies such as these have indicated that bacterial components or metabolites are a promising strategy for prevention and treatment of allergic asthma due to their high bioavailability, easy assessment of dose–response relationships and low variability of clinical responses [10]. Flagellin, D-tryptophan, bacterial DNA, and some of *S. pneumoniae* components have been shown to suppress allergic asthma in mice [11–15].

*S. pneumoniae* PepN, is associated with reduced activation of the immunomodulator ZAP-70 and the p38—ERK1/2 MAP kinase signaling pathway [16]. Our previous results indicated that PepN administered during sensitization could suppress OVA-induced allergic asthma, partially mediated by reducing the recruitment of lung CD11b^+^ dendritic cells and the release of IL-33. Furthermore, we observed a consistent inhibitory effect of PepN treatment prior to sensitization [17]. These data indicated that PepN is a potential prevention and treatment strategy for allergic asthma.

Macrophages play key roles in homeostasis, tissue remodeling, and host defense in lung [18, 19]. In particular, alveolar macrophages (AMs) are intimately involved in the development and progression of asthma via allergic antigen presentation, removal of apoptotic cells, producing pro-inflammatory or anti-inflammatory cytokines as appropriate and regulate the cross talk between immune cells [18, 20–24]. Therefore, AMs are regarded as an effective therapeutic target in asthma [20]. Macrophages also undergo metabolic reprogramming during their activation to maintain tissue homeostasis that further modulate their phenotypes and functions. For instance, pro-inflammatory or anti-inflammatory macrophages choose distinct metabolic pathways during activation to meet their demands for energy and production of specific function-associated cytokines [25]. For example, glycolysis and remodeling of the tricarboxylic acid cycle support the inflammatory phenotype of macrophages while fatty acid oxidation (FAO) and OXPHOS are associated with anti-inflammatory response [26–30]. Mitochondrial ATP synthesis is performed by the OXPHOS system but this process also generates reactive oxygen species (ROS) from electron leakage during electron transport that react prematurely with oxygen [31]. Low levels of ROS are necessary for inducing cell adaptation although excessive production in macrophages can be mutagenic for mitochondrial DNA and induce a pro-inflammatory phenotype resulting in the synthesis and release of IL-1β and allergic asthma development [32]. Therefore, reprogramming the metabolic pathways of macrophages to acquire an anti-inflammatory phenotype or blocking inflammatory memory of macrophages may be an effective remedy to prevent asthma development.

In the current study, we used a mouse model of allergic asthma and pretreated mice with the PepN protein in an attempt to develop an anti-inflammatory response from macrophages. PepN stimulated the recruitment of bone marrow derived macrophage (BMDM) to the respiratory tract and altered their phenotype to CD11c^high^ and a metabolic preference for OXPHOS. This process enabled the macrophages to acquire an anti-inflammatory property and also upregulated costimulatory molecules to promote Treg induction that further suppressed allergic airway inflammation.

## Methods

### Primers in quantitative PCR are included in Additional file 1: Table S1

#### Materials

ELISA kits used in this study were IL-4 (Biolegend, Cat # 431104), IL-5 (Biolegend, Cat # 431204), IL-13 (R&D Systems, Cat # DY413-05), TNF-α (Biolegend, Cat # 430904), IL-1β (Biolegend, Cat # 432604), IL-10 (Biolegend, Cat # 431414), IL-6 (Biolegend, Cat # 431304), CCL2 (R&D Systems, Cat # DY479-05), Complex I ELISA Kit (Meimian, China, Cat # MM-44745M2), ATP ELISA Kit (Meimian, China, Cat # MM-43789M2).

Antibodies used in this study were Caspase-8 (R&D Systems, Cat # AF705-SP), Cleaved Caspase-3 (CST, Cat # 9664), β-actin (Affinity, China, Cat # ab208670), AMPKα (D5A2) (CST, Cat # 5831), p-AMPKα(Thr172) (CST, Duet # 8208), Stat3 (124H6) (CST, Cat # 9139), p-Stat3 (Tyr705) (CST, Cat # 9145), p-NF-κB p65 (CST, Cat # 3033T), PD-L1 (Abcam, Cat # ab213480), IL-1 beta (Abcam, Cat # ab254360), Goat monoclonal anti-mouse-HRP (Biosharp, China, Cat # 21102333), Mouse monoclonal anti-mouse-HRP (Biosharp, China, Cat # 21183464).

Busulfan (Otsuka, Cat # 1E049A), Oligomycin (Sangon Biotech, Cat # 1404-19-9), FCCP (Macklin, Cat # C867805), Rotenone (Macklin, Cat # R817233), Antimycin A (Maokang Biotechnology, Cat # 1397-94-0), Glucose (Macklin, Cat # G6172), 2-DG (Sangon Biotech, Cat # 154-17-6), Red blood cell lysis buffer (Biosharp Life science, Cat # BL503A), DNase I (Beyotime Biotechnology, Cat # D7073), Collagenase IV (Solarbio, Cat # C8160), Fixative solution (4% formaldehyde, methanol-free) (Biosharp Life science, Cat # BL539A).

THP-1 (National collection of Authenticated cell cultures, TCHu 57).

#### Mice

Wild-type BALB/c, C57BL/6 were purchased from the Animal Experimental Center of Chongqing Medical University. CD45.1 mice were obtained from the Children's Hospital of Chongqing Medical University. All experimental mice were exclusively female, aged between 6 to 8 weeks. All mice were meticulously maintained in a specific pathogen-free condition, under the supervision of Laboratory Animal Experimental Center at Chongqing Medical University. The execution of all animal experiments adhered to the ethical guidelines established by the Institutional Animal Care and Use Committee at Chongqing Medical University.

### Asthma model and PepN experimental therapies

To induce asthma model, BALB/c mice were sensitiazation with 1 μg HDM (Greer Laboratories, cat#XPB82D3A25) in 30 μl of sterile phosphate-buffered saline (PBS) by the intranasal (i.n.) route on days 0, the followed challenge with 10 μg HDM in 30 μl PBS given i.n. from days 7 to 11. The control group mice received the same treatment, with PBS being administered at equivalent time points.For PepN treatment prior to sensitization, mice were subjected to anaesthesia and received intranasal instillation of a 30 μl solution containing 50 μg of PepN on days -8, -5 and -2. Subsequently, the modeling protocol was consistent with that of the HDM group. On day 14, all mice were euthanized in a humane manner via intraperitoneal injection of a 1.5% pentobarbital sodium solution.

### CD11c^high^ macrophages depletion and transfer

To deplete CD11c^high^ macrophages in lungs, PepN + HDM or HDM treatment mice received intranasally administration of 50 μl of clodronate liposomes (Formumax Scientific, Cat # C-005) on days 5 and 9. CD45^+^F4/80^+^CD11c^high/int/−^ cells and Foxp3^+^Treg were isolated using FACSCantoTM (BD Biosciences, Franklin, NJ, USA).

To validate the anti-inflammatory impact of CD11c^high^ macrophages, a total of 1.25 × 10^5^ CD45^+^F4/80^+^CD11c^high^ cells, sorted from the lungs of PepN + HDM treatment mice using FACSAriaTM II, were adoptively transferred intraperitoneally into mice on day 6 of the HDM-induced asthmatic model. Subsequently, all mice were sacrificed for analysis 72 h after the final challenge. The inflammatory profile was assessed through ELISA and histochemistry staining.

### Assessment of cell proliferation using EdU incorporation

For in vivo EdU incorporation, BALB/c mice received three separate administrations of 50 μg of PepN on days 1, 4 and 7. Macrophages were intraperitoneally injected (i.p.) with EdU (15 mg/kg) 24 h before each of these time points. On day 8, the mice were euthanized, and lung single-cell suspension were prepared. The harvested cells were subjected to surface staining using F4/80 (Biolegend, Cat # 123110) and CD11c (Biolegend, Cat # 117306). Intracellular EdU staining was carried out in accordance with the manufacturer’s instructions using the EdU flow kit (RiboBio, Cat # C10371-2). Subsequently, the extent of EdU incorporation in macrophages was quantified.

### Bone marrow derived macrophages isolation and culture

For isolation of BMDMs from C57BL/6 J mice between the ages of 6–8 weeks, tibias and femurs were moved by sterile techniques and bone marrow was flushed with DMEM medium. Cells were plated in medium supplemented with 10 ng/ml M-CSF (Sino Biological, Cat # 51,112-MNAH), 10% Fetal Bovine Serum (Ausbian), 1% Pen/Strep (Biosharp Life science, Cat # BL505A), And were maintained for 7 days at 37℃ in a humidified atmosphere of 5% CO_2._ Fresh differentiation medium was replaced every 2 days.

### BMDMs adoptive transfer model

To establish the BMDMs transfer mice model, a total of 4 × 10^5^ viable PepN-pretreated BMDMs were adoptively transferred to the HDM treatment mice via intranasal administration 1 day before the first challenge. All mice were euthanized on day 14.

### Broncho-alveolar lavage (BAL) fluid

Following euthanasia, a tracheal catheterization procedure was conducted, and bronchoalveolar lavage (BAL) fluid was obtained through two sequential lung flushes using 0.5 ml of pre-cooled sterile PBS. Subsequently, cells within the BAL fluid were quantified using a modified Neubauer Counter. Additionally, Wright's staining was employed to perform a differential cell count analysis on the BAL fluid. Flow cytometry was utilized to determine the specific subgroups of macrophages, and cytokine production in cell-free supernatants was assessed using dedicated enzyme-linked immunosorbent assay (ELISA) methodologies. Protein concentration and viral titers were also determined, while supernatants were collected for the evaluation of cytokines/chemokines levels.

### RNA extraction, RT-PCR and qPCR

Total RNA from lung tissues or macrophages using RNAiso Plus reagent (TaKaRa, Cat # 9108), and reverse-transcribed using a PrimeScript™ RT reagent Kit (TaKaRa, Cat # RR037A) according to the manufacturer's protocol. Quantitative real-time PCR (qRT-PCR) was performed with the SYBR Green Master Mix (TsingkeBiotechnologyCo., Ltd., Cat # TSE202) cDNA and specific primers. The expression of each gene was normalized to that of *β-actin*. Sequences of primers used in the studies are provided in the Additional file 1: Table S1.

### Flow cytometry and intracellular protein staining

For the generation of single lung cell suspensions, the lungs were initially perfused with PBS and subsequently dissected into 1mm^3^ pieces. These tissue fragments underwent a 10-minutedigestion process at 37 °C in a medium containing 1 mg/mL of type IV collagenase and 5 U/mL DNase I. After digestion, the sample were filtered, and erythrocytes were lysed. Non-specific antibody binding sites were blocked by incubating the cells with anti-CD16/32 (Biolegend, Cat # 101302).

For cell surface marker staining, the macrophages were labeled with specific antibodies: APC-conjugated anti-CD45 (Biolegend, Cat # 103112), PE-conjugated anti-F4/80 (Biolegend, Cat # 123110), FITC-conjugated anti-CD11c (Biolegend, Cat # 117306), APC-conjugated anti-PD-L1 (Biolegend, Cat # 124311) and PE-conjugated anti-CD80 (Biolegend, Cat # 12-0801-81).

Intracellular protein staining was conducted using a Fixation/Permeabilization solution as per the manufacturer’s guidelines. Tregs were stained with specific antibodies: BV605-conjugated anti-CD4 (Biolegend, Cat# 116027), FITC-conjugated anti-CD25 (Biolegend, Cat # 102006) and PE-conjugated anti-Foxp3 (Biolegend, Cat # 126404).

Cell proliferation was assessed using an EdU staining kit. Cell phenotyping and sorting were carried out using BD FACSCanto plus and FACSCanto II (BD Biosciences, Franklin, NJ, USA). Data analysis was performed using FlowJo software.

### Hoechst nuclei staining

Alveolar macrophages were harvested from both the asthma model and control group, and seeded at a density of 1 × 10^5^ cells/well per will into 6-well plates. Cells were fixed with 4% paraformaldehyde for 15 min and subjected to staining with Hoechst 33342 (Beyotime Biotechnology, Cat # C1027) for 10 min at RT. Following, cells were washed twice with PBS, and apoptotic morphology were examined under Leica DMI8 confocal microscope (Wetzlar, Germany).

### Apoptosis detection

Alveolar macrophages were obtained from mice in both the PepN + HDM and HDM treatment groups. Cells were washed using pre-cooled PBS and resuspend in 1 × Binding Buffer. To assess cell apoptosis, an Annexin V FITC apoptosis detection kit (BD pharmingen, Cat # 556547) was employed following the manufacturer’s instructions.

### Bone marrow chimera

To investigate the origin of CD11c^high^ macrophages in the lung of PepN + HDM treatment mice, Bone marrow chimeras were established, 5 × 10^6^ bone marrow cells isolated from C57BL/6 mice (expressing CD45.1 alloantigen) were transferred into 8-wk-old recipient mice (expressing the CD45.2 alloantigen) that has been lethally irradiated (administered a single dose of 5 Gy γ-radiation for twice using a 225 kV X-RAY irradiator; Radsourse). To protect tissue-resident CD11c^high^ macrophages in lung from radiation, we constructed a bone marrow chimera with thoracic shielding by using lead shield that covered the lungs during irradiation, distinguishing between CD11c^high^ macrophages and CD11c^int^ macrophages. To eliminate peripheral blood monocytes within the shielded region of recipient mice, we administrated the myeloablative agent busulfan 6 h after irradiation, followed by bone marrow infusion 12 h later. Mice were maintained on antibiotics (trimethoprim/sulfamethoxazole.) for 4 weeks after bone marrow transfer. Subsequently, the bone marrow chimeras were employed for experiments 8 weeks post-hematopoietic reconstitution.

### RNA-seq

CD11c^high^ macrophages were isolated from lung tissue using flow cytometry, total RNA was extracted with Trizol Kit following the manufacture’s protocol. And perform subsequent library preparation and sequencing at Novogene Bioinformatics Technology Co.Ltd. (Beijing, China). Agilent 2100 Bioanalyzer (Agilent Technologies, Palo Alto, California, USA) was used to identify RNA-Seq libraries to assess the integrity of the RNA. cDNA libraries were prepared using NEBNext® Ultra™ RNA Library Prep Kit for Illumina®. The quality of cDNA libraries was measured by Qubit 2.0 fluorometer DNA assay kit and submitted for sequencing (Agilent 2100 Bioanalyzer, California, USA). Differential expression analysis of two conditions/groups (three biological replicates per condition) was performed using the DESeq2R package (1.20.0). DESeq2 provides statistical routines based on a negative binomial distribution model for determining differential expression in digital gene expression data. The resulting P-values were adjusted using the Benjamini and Hochherg's approach for controlling the false discovery rate. Genes with an adjusted P-value < 0.05 found by DESeq2 were assigned as differentially expressed, and absolute foldchange of 2 was used as the threshold for significantly differential expression.

### Cell oxygen consumption rate and Glycolysis assay analysis

Alveolar macrophages were isolated from bronchoalveolar lavage fluid (BALF) obtained from mice in PepN + HDM and HDM treatment group. Cells were seeded into a 96-well culture plate, the assessment of oxygen consumption and glycolysis was measured by commercial detect kits (Abcam, Cat # ab197243; Abcam, Cat # ab197244) according to the manufacturer's instructions using a Varioskan LUX (Thermo Fisher).

### Transmission electron microscopy

Alveolar macrophages were fixed in 3% glutaraldehyde, following by post-fixation in 1% osmium tetroxide. Dehydration was then carried out in a series of acetone solution. Subsequently, the cells underwent embedding and sectioning for transmission electron microscopy (JEM-1400-FLASH). Assessment of mitochondrial morphology was performed, with distinctions made between normal and abnormal features. Aberrant mitochondria were characterized by the presence of specific structural anomalies, including the loss of cristae, reduced electron density in the matrix, compromised mitochondrial membrane integrity, and the formation of autophagosomes structures.

### Confocal microscopy

For labeling mitochondria, cells were cultured overnight on glass coverslips and incubated with 400 nM Mito Tracker Deep Red (ThermoFisher Scientific, Cat# M22426) for 40 min at 37 °C. After staining is complete wash cells with fresh prewarmed PBS for twice, and then stained with Hoechst 33342 for 10 min at RT. For labeling mt-ROS, cells were incubated with 5 μM Mito SOX Red mitochondrial superoxide indicator (ThermoFisher Scientific, Cat # M36008) in the dark for 10 min at 37 °C. Afterward, cells were washed with PBS for 3 times to remove the dye and then stained with Hoechst 33342 for 10 min at RT. Moreover, the images were obtained using a Leica DMI8 confocal microscope.

### Mitochondrial ROS and mitochondrial membrane potential measurement

Alveolar macrophages were isolated from BALF, cells were incubated with 5 μM Mito SOX Red mitochondrial superoxide indicator and 400 nM Mito Tracker Deep Red at 37 °C 5% CO_2_, respectively. Cells were washed twice with prewarmed PBS. Subsequently, mt-ROS and mitochondrial membrane potential for cells were analyzed by flow cytometry.

### Isolation of CD4^+^T cells and co-culture with macrophages

Alveolar macrophages were harvested from BALF and subsequently seeded at a concentration of 2 × 10^5^ cells per well in 96-well plates. Simultaneously, naïve CD4^+^T cells were isolated from the spleen through immunomagnetic separation using beads (Miltenyi Biotec, Cat # 130–104-453) and were seeded at a density of 1 × 10^6^ cells per well in 96-well plates. The culture medium employed was supplemented with 5 μg/mL of anti-CD3e (eBioscience, Cat # 16-0031-81) and soluble anti-CD28 (eBioscience, Cat # 16-0281-81). Following this, a co-cultured of these cells was maintained for an additional 3 days within a standard set at 37°Cwith 5% CO_2_.

### Statistical analysis

Statistical significance was performed using the analysis of variance (ANOVA) or a two-tailed Student’s t test. All analyses were performed using GraphPad Prism version 9.00 (GraphPad). Data are shown as means ± SD. P < 0.05 was considered significant.

## Results

### PepN-pretreatment inhibits HDM-induced allergic airway inflammation and increases the number of F4/80^+^CD11c^high^ macrophages in mice lungs

**Fig. 1:**
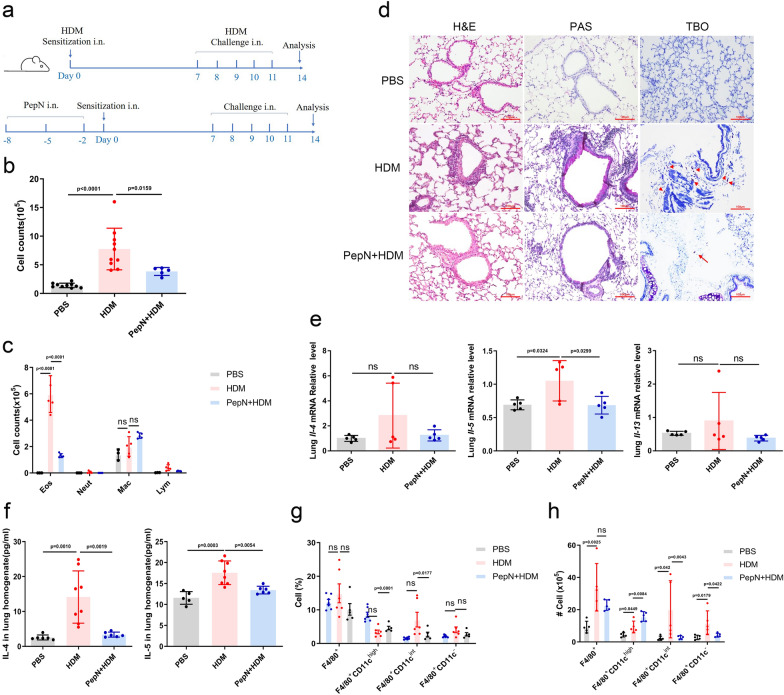
PepN-pretreatment inhibits HDM-induced allergic inflammation and increases CD11c^high^ macrophage levels. **a** Experimental design in BALB/c mice. Mice were intranasally instilled with PepN protein (50 μg in 30 μl PBS). Two days after treatment, mice were sensitized with 1 μg HDM (day 0) and challenged with 10 μg HDM (days 7 to 11) i.n.. **b, c** The number of total inflammatory cells (**b**) and differential cell counts (**c**) in BALF (n = 3–10). **d** Microscopic images of H&E-, PAS- and toluidine blue-stained (TBO) lung tissue sections of mice (200 × magnification), mast cells were shown by arrows. **e** mRNA relative level of Th2 cytokines in lung tissues. **f** ELISA of Th2 cytokines in lung homogenate (n = 5–8). **g**, **h** Percentage (**g**) and absolute number (**h**) of macrophages (F4/80^+^CD11c^high/int/−^) in mice lungs were measured by flow cytometry (n = 5–7). Statistical analysis by one-way ANOVA with Tukey’s multiple-comparison test (**b, c** and **e–h**). Data are represented as mean ± SD (**b**, **c** and **e–h**). ns, not significant

In this study, we pretreated mice with PepN and developed an HDM-induced asthma model to further verify its effect on asthma (Fig. 1a). PepN-pretreated mice exposed to HDM (PepN + HDM group) displayed reduced levels of eosinophils, less peribronchial and perivascular inflammatory cell infiltration and an inhibition of goblet-cell hyperplasia and mast cell degranulation (Fig. 1b–d). These mice also possessed decreased levels of type 2 inflammatory cytokines in their lungs relative to those of HDM-induced mice (HDM group) (Fig. 1e, f). Interestingly, even though macrophages play important roles in asthma, the number of macrophages in BALF did not significantly differ between the PepN + HDM and HDM groups (Fig. 1c). We therefore analyzed macrophage subpopulations in lungs using flow cytometry, and F4/80^+^CD11c^high^, F4/80^+^CD11c^int^ and F4/80^+^CD11c^−^ represent TR-AMs, Mo-AMs and IMs, respectively [33, 34]. PepN + HDM mice displayed significantly increased numbers of F4/80^+^CD11c^high^ macrophages in lungs than those of HDM mice and reduced numbers of CD11c^int^ and CD11c^−^ macrophages (Fig. 1g, h) Additional file 1: Fig. S1a). Taken together, these data demonstrated that PepN-pretreatment significantly suppresses airway inflammation in HDM-induced asthmatic mice and this is most likely linked to these alterations in the macrophage subpopulations. Therefore, we focused on the effect that PepN displayed on the appearance of CD11c^high^ macrophages during the inhibition of allergic asthma.

### PepN-pretreatment inhibits the allergic airway inflammation through F4/80^+^CD11c^high^ macrophages

**Fig. 2:**
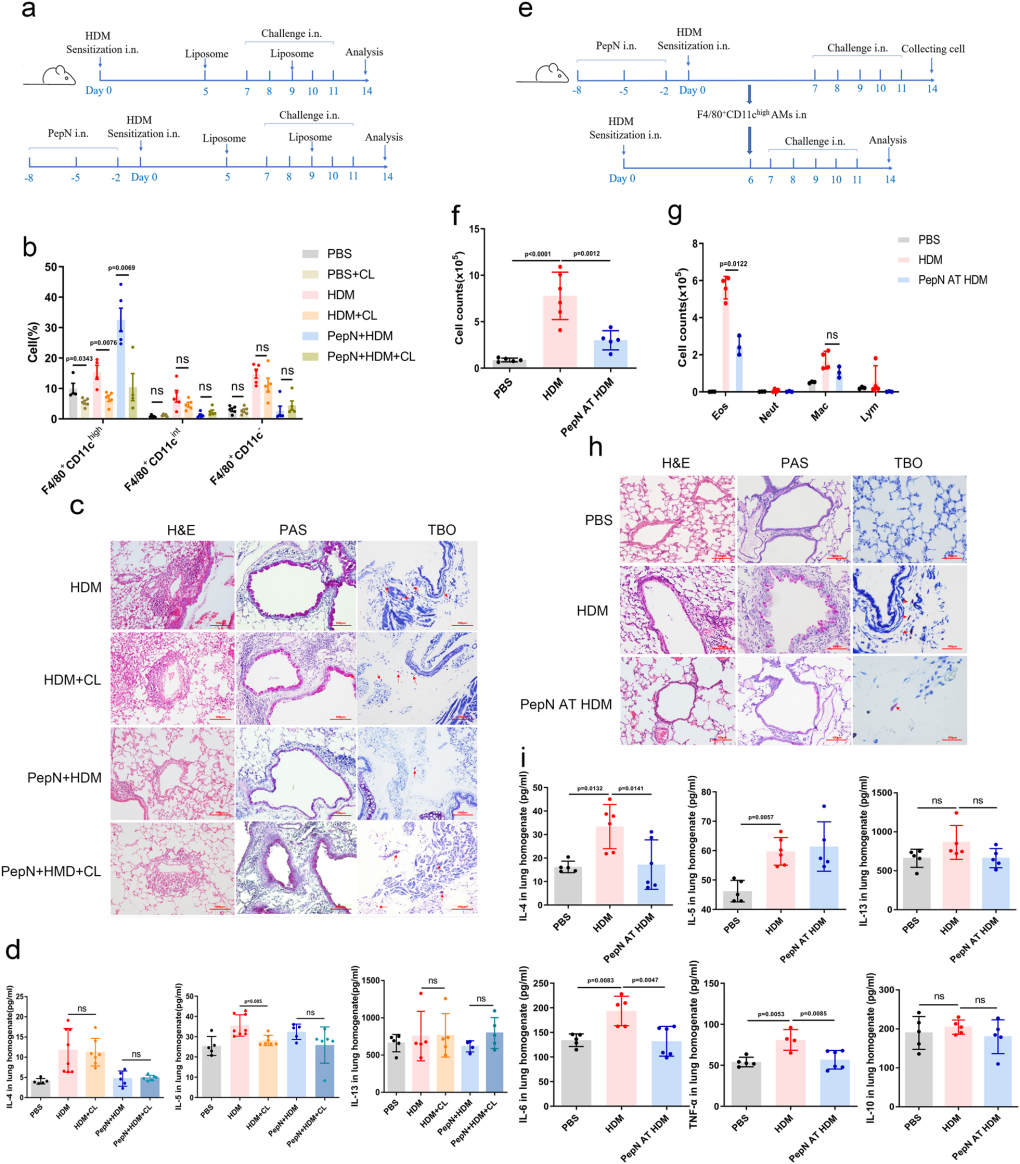
PepN-pretreatment attenuates airway inflammation via F4/80^+^CD11c^high^ macrophages. **a** Experimental design using BALB/c mice pretreated with PepN (50 μg) and PBS, HDM, 50 μl clodronate liposomes (CL) as indicated. **b** Flow cytometry analysis of % macrophages (F4/80^+^CD11c^high/int/−^) in lungs of mice intranasally treated with clodronate liposomes (n = 4–5). **c** Microscopic images of stained lung tissue sections from mice as indicated (200 × magnification). **d** ELISA of Th2 cytokines in lung homogenates (n = 5–8). **e** Experimental design of adoptive transfer (AT) experiments using HDM-treated BALB/c recipient mice given FACS-sorted PepN-pretreatment CD11c^high^ macrophages (1.25 × 10^5^/mice) on day 6 following sensitization. **f**, **g** Total inflammatory cells (**f**) and differential cell counts (**g**) in BALF (n = 3–6). **h** Microscopic images of stained lung tissue sections of mice as indicated (200 × magnification). **i** ELISA of cytokines in lung homogenates (n = 4–6). Statistical analysis by a two-tailed student’s t test (**b**, **d**), one-way ANOVA with Tukey’s multiple-comparison test (**f, g** and **i**). Data are represented as mean ± SD (**b**, **d**, **f**, **g** and** i)**. ns, not significant

To explore whether the inhibitory effect of PepN on airway inflammation in allergic asthma was associated with CD11c^high^ macrophages, we depleted this macrophage population from HDM and PepN + HDM treated mice by intranasal administration of clodronate liposomes on day 5 and 9 following sensitization (Fig. 2a). We found that clodronate liposomes administration specifically depleted CD11c^high^ macrophages in lungs with no impact on CD11c^int^ and CD11c^−^ macrophages (Fig. 2b). Interestingly, HDM mice treated with clodronate liposome displayed fewer peribronchial and perivascular inflammatory cells, less goblet cell proliferation and lower expression of the Th2 cytokine IL-5 than did the untreated control (Fig. 2c, d). However, PepN + HDM mice treated with clodronate liposome showed severe inflammatory infiltration in the lungs, more mucus production in airways and higher expression of the Th2 cytokine IL-13 (no statistical difference) than controls (Fig. 2c, d). These results indicated that CD11c^high^ macrophages in the HDM group and the PepN + HDM group possessed differing functions, i.e., CD11c^high^ macrophages (HDM group) displayed a pro-inflammatory profile while CD11c^high^ macrophages (PepN + HDM group) displayed an anti-inflammatory profile.

To further verify this results, CD45^+^F4/80^+^CD11c^high^ macrophages from lungs of PepN + HDM mice were sorted and adoptively transferred (1.25 × 10^5^ cells) into HDM-sensitized mice (Fig. 2e). The transferred CD11c^high^ macrophages alleviated the recruitment of eosinophils and mast cells, suppressed the expression of *Il-4, Il-6* and *Tnf-α* and reduced the infiltration of inflammatory and goblet cells (Fig. 2f–i). These results indicated that the expanded CD11c^high^ macrophages of PepN-pretreatment are indispensable for the suppression of the development of airway inflammation.

### PepN-pretreatment alters proliferation and apoptosis of F4/80^+^CD11c^high^ macrophages

**Fig. 3:**
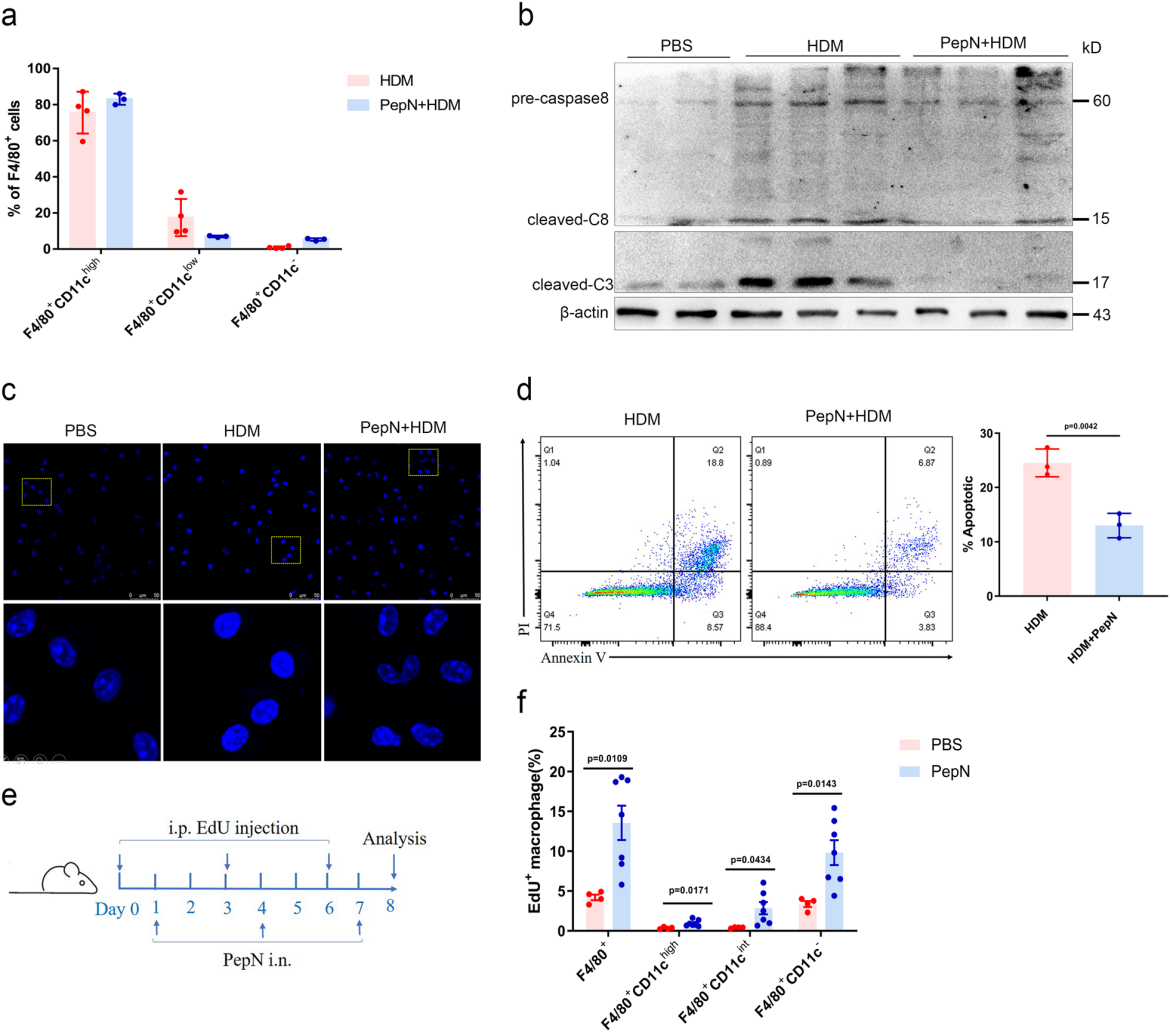
PepN-pretreatment alters proliferation and apoptosis of CD11c^high^ macrophages. **a** AM (%) of F4/80^+^CD11c^high/int/−^ in BALF (n = 3–4). **b** AM caspase8 and caspase3 protein levels in BALF measured using Western blotting (n = 2–3). **c** Apoptosis levels based on the quantification of nuclei in Hoechst 33,242-stained cells. Scale bars, 50 μm. **d** AM apoptosis in BALF analysed using flow cytometry (n = 3). **e**, **f** Mice were injected intraperitoneally with EdU 24 h before day 1, 4 and 7 intranasal administrations of PepN, EdU^+^ macrophages (%) were assessed on day 8. Statistical analysis by a two-tailed student’s t test (**d**, **f**). Data represent one of at least five samples from three independent experiments (**b**). Data are represented as mean ± SD (**a**, **d** and **f**)

Given the important role that CD11c^high^ macrophages of PepN + HDM mice played in the protection against airway inflammation, we sought to determine the origin of these increased macrophages. We found that CD11c was highly expressed in nearly 80% of F4/80^+^ cells in BALF in both PepN + HDM mice and HDM mice (Fig. 3a, Additional file 1: Fig. S1b). Therefore, these macrophages were used as the source of CD11c^high^ macrophages in subsequent experiments.

Cell apoptosis and proliferation are opposing factors that directly affect cell numbers. We therefore quantified AMs apoptosis and proliferation in mice following PepN-pretreatment. PepN-pretreatment inhibited the activation of apoptosis-related proteins caspase-8 and caspase-3 (Fig. 3b). Chromatin condensation of AMs in the HDM group was also more intense than for the PepN + HDM group and there were no obvious signs of nuclear fragmentation (Fig. 3c). Annexin V-propidium iodide (PI) staining also indicated that PepN-pretreatment significantly decreased AMs apoptosis (Fig. 3d).

Under physiological conditions, AMs can self-renewal in situ so we examined whether PepN can modulate proliferation of CD11c^high^ macrophages. We administrated mice with PepN alone at different time points and analyzed the incorporation of EdU by macrophages in vivo. PepN exposure increased the percentage of EdU^+^CD11c^high^ macrophages, and similar findings were observed in CD11c^int^ and CD11c^−^ macrophages (Fig. 3e, f, Additional file 1: Fig. S2a). These results indicated that PepN can increase the numbers of CD11c^high^ macrophages partly by reducing apoptosis and promoting proliferation.

### PepN-pretreatment increased CD11c^high^ macrophages partially derive from CD11c^int^ macrophages

**Fig. 4:**
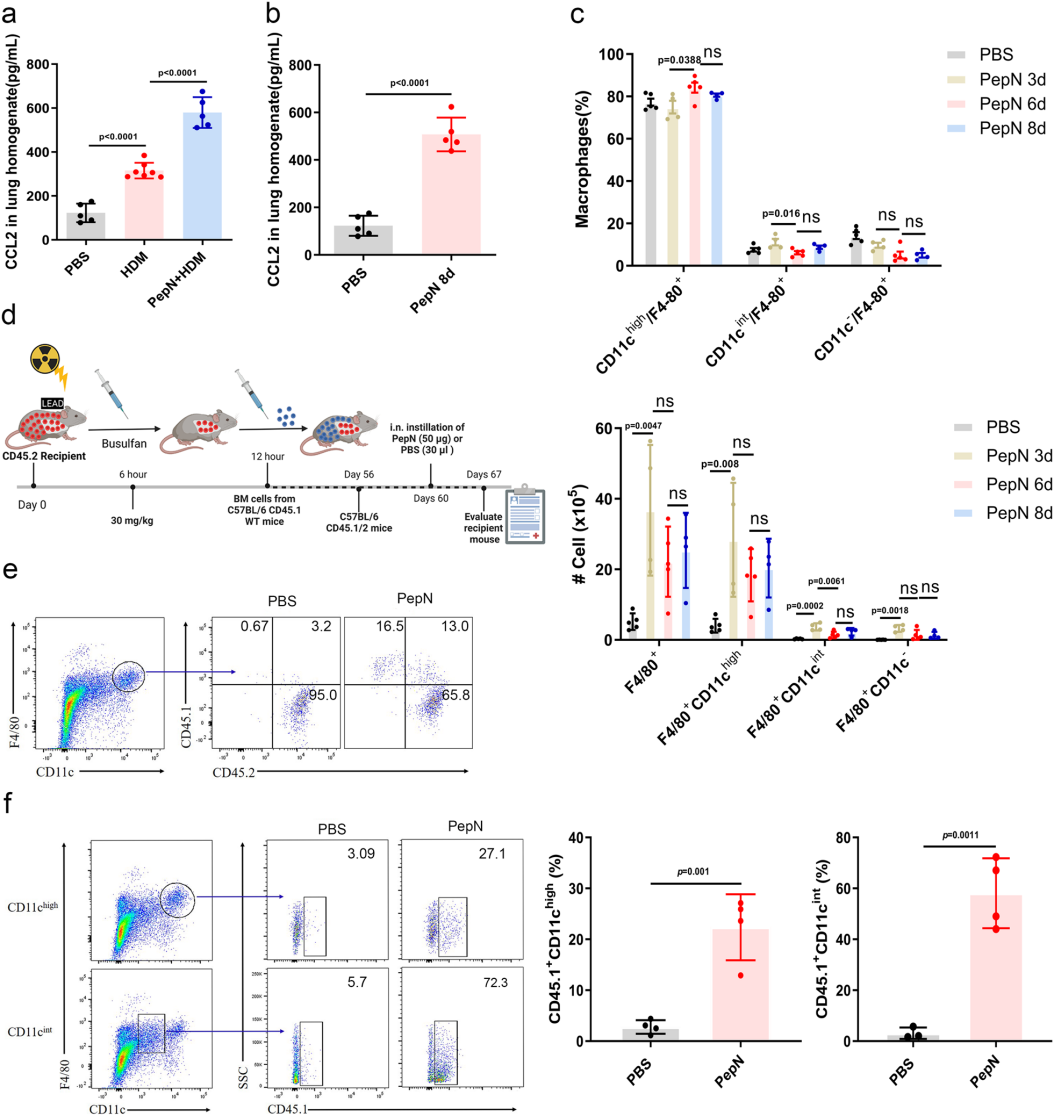
PepN-pretreatment expands CD11c^high^ macrophages that were partially derived from CD11c^int^ macrophages. **a**, **b** ELISA of cytokine CCL2 in lung homogenates (n = 5–7). **c** Percentage and absolute number of macrophages (F4/80^+^CD11c^high/int/−^) in lungs of mice treated with PepN on days 0, 3 and 6. Macrophage staining was quantified by flow cytometry two days following each treatment (n = 4–5). **d** Schematic diagram of the generation of mice bone marrow chimeras with thoracic shielding and busulfan and PepN-treatments as indicated. **e**, **f** Representative dot plots reflecting BM-derived (%) and tissue-resident (%) macrophages in the lung of chimeric mice 8 weeks after irradiation and bone marrow transfer and 7 days after i.n. PepN treatment (50 μg). Statistical analysis by a two-tailed student’s t test (**b**), one-way ANOVA with Tukey’s multiple-comparison test (**a**–**c**). Data pooled from at least 3–4 independent experiments (**e**, **f**). Data are represented as mean ± SD (**a**–**c**). ns, not significant

Mo-AMs are primarily F4/80^+^CD11c^int^ and this cellular pool is maintained and expanded from CCR2-mediated recruitment and differentiation of local BMDMs [35]. Therefore, we explored the effects of PepN on the BMDM population. The lungs of PepN + HDM mice displayed higher levels of the macrophage chemokine CCL2 compared with both the HDM and PBS mouse groups (Fig. 4a). We also observed that the CCL2 expression increased following PepN treatment versus the PBS controls (Fig. 4b). This indicated that PepN can recruit monocytes into the lung tissues of mice. Moreover, as previously mentioned, PepN treatment also promoted proliferation of CD11c^int^ macrophages (Fig. 3f). However, PepN treatment significantly decreased the numbers of CD11c^int^ macrophages in lungs while simultaneously increasing the numbers of CD11c^high^ macrophages (Fig. 1g).

Monocyte-derived macrophages can be converted into AMs [4], so these data implied that CD11c^int^ macrophages were altered to the CD11c^high^ phenotype with PepN treatment. To confirm this hypothesis, we counted CD11c^high/int/−^ macrophages in lungs at days 3, 6 and 8 following PepN administration. We found that the number of CD11c^−^ macrophages did not change significantly during this time course. However, the proportion and numbers of CD11c^int^ macrophages increased on day 3 and decreased on days 6 and day 8 following PepN treatment. In contrast, the proportion and numbers of CD11c^high^ macrophages increased and peaked at days 6 and 8 (Fig. 4c, Additional file 1: Fig. S2b). These results demonstrated that the increased CD11c^high^ macrophages by PepN were partially derived from CD11c^int^ macrophages.

To further confirm whether these recruited monocytes (CD11c^int^) were altered to CD11c^high^ macrophages, we transfered CD45.1 bone marrow cells into sub-lethally irradiated CD45.2 mice that were thorax-shielded to protect TR-AMs from radiation. They were then administered the myeloablative agent busulfan to delete the residual recipient bone marrow population from the shielded region (Fig. 4d). The bone marrow chimeras were treated with PepN (50 μg) 7 weeks after transplantation and 27.1% of CD11c^high^ macrophages were of donor origin. In contrast, only 3.09% of CD11c^high^ macrophages were of donor origin in the PBS control mice (Fig. 4e, f). These data indicated that PepN treatment can recruit monocytes from the bone marrow and convert them to CD11c^high^ macrophages.

### PepN-pretreatment preprograms the mitochondrial OXPHOS bias of CD11c^high^ macrophages resulting in an anti-inflammatory phenotype

**Fig. 5:**
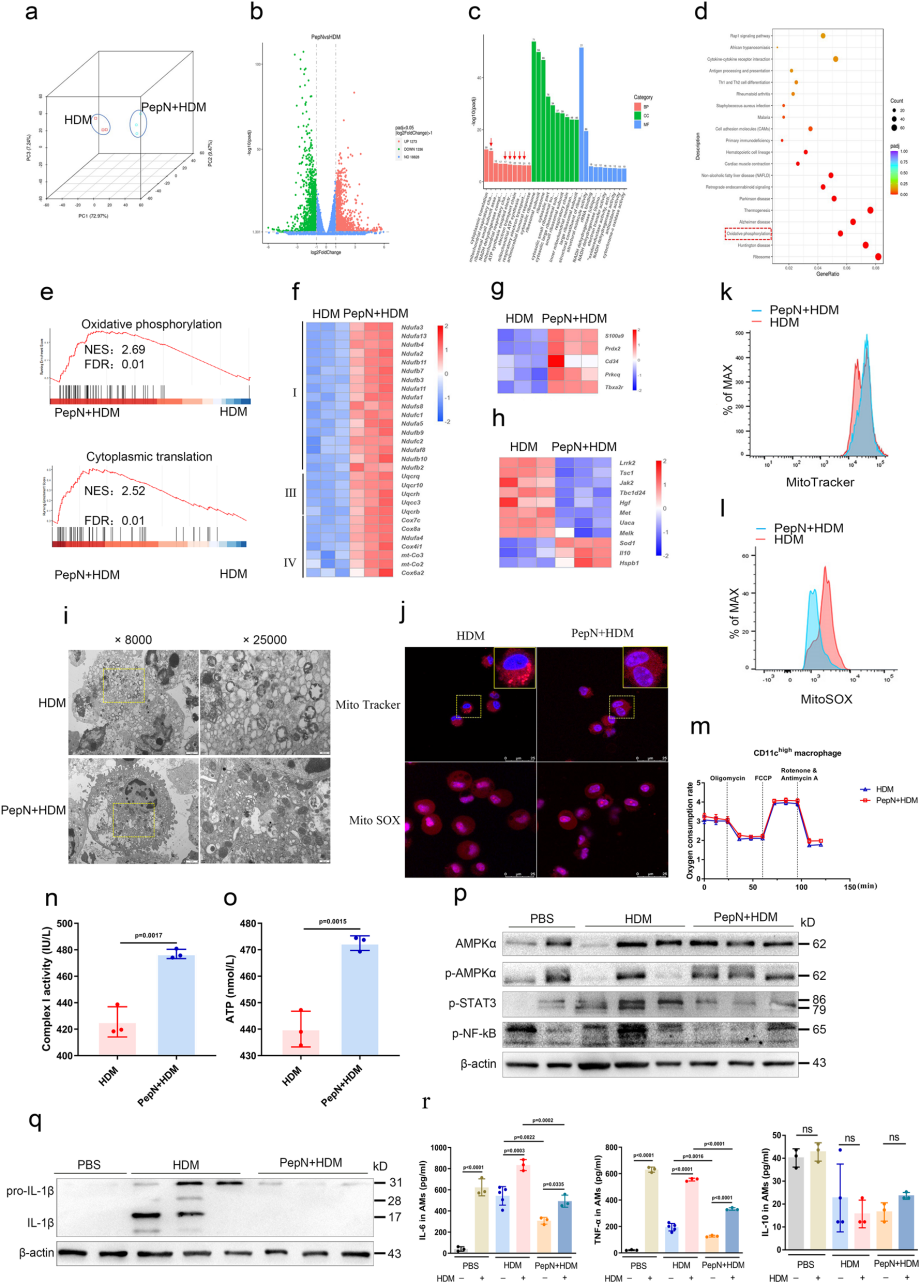
PepN-pretreatment preprograms CD11c^high^ macrophages for an OXPHOS preference and an anti-inflammatory phenotype. **a** Principal component analysis of RNA-seq data of CD11c^high^ macrophages. **b** Genes significantly (p adjusted < 0.05) up-regulated and down-regulated are depicted in red and green, respectively. **c** GO biological process enrichment results. **d** KEGG pathway enrichment results. **e** Gene set enrichment analysis of the RNA sequencing data. **f–h** Heat map diagrams of relative expression levels of mitochondrial respiratory chain complex-related genes (**f**), positive regulation of wound healing-related genes (**g**) and cell death in response to oxidative stress-related genes (**h**). **i** Transmission electron photomicrographs depicting mitochondrial morphology of CD11c^high^ macrophages. Scale bars, 2 μm and 500 nm. **j** Mitochondrial morphology (Mito Tracker) and mt-ROS production (Mito SOX) of CD11c^high^ macrophages observed using confocal microscopy. Scale bars, 25 μm. **k**, **l** Mitochondrial membrane potential (**k**) and mt-ROS (**l**) of CD11c^high^ macrophages were detected by flow cytometry. **m** Oxygen consumption rate (OCR) of CD11c^high^ macrophages with indicated treatments (n = 3). **N** Enzymatic activities of mitochondrial complex I of CD11c^high^ macrophages (n = 3). **o** ATP production by CD11c^high^ macrophages (n = 3). **p** Activation of AMPK, STAT3 and NF-κB signaling pathway in CD11c^high^ macrophages were detected by Western blotting. **q** IL-1β levels in CD11c^high^ macrophages measured by Western blotting. **r** Cytokines in supernatants of CD11c^high^ macrophages after restimulation for 1 d ex vivo with HDM (1 μg/ml) were measured by ELISA. Statistical analysis by a two-tailed Student’s t test (**n**, **o**) or one-way ANOVA with Tukey’s multiple-comparison test (**r**). Data represent one of at least five samples from three independent experiments (**p**, **q)**. Data are represented as mean ± SD (**n**, **o** and **r**). ns, not significant

To define the function of CD11c^high^ macrophages in inhibiting allergy airway inflammation after PepN-pretreatment, RNA sequencing was performed on sorted CD11c^high^ macrophages from the lungs of PepN + HDM mice and HDM mice. The gene expression profiles of CD11c^high^ macrophages were clearly distinct between the two groups (Fig. 5a) and included 1273 up-regulated and 1336 down-regulated differentially expressed genes (DEGs) (Fig. 5b). GO analysis revealed these DEGs were enriched in biological processes including mitochondrial respiratory chain complex assembly, NADH dehydrogenase and electron transport chain (Fig. 5c). They were also enriched in KEGG pathway of OXPHOS metabolism (Fig. 5d). Gene set enrichment analysis (GSEA) also revealed up-regulated gene expression for mitochondrial OXPHOS and cytoplasmic translation genes in CD11c^high^ macrophages of the PepN + HDM group (Fig. 5e).

We further analysed the DEGs of CD11c^high^ macrophages in PepN + HDM and HDM group. The PepN + HDM group (relative to the HDM group) displayed enhanced expression of mitochondrial electron transport chain (Fig. 5f) and wound-healing genes (Fig. 5g) and reduced cell death expression genes in response to oxidative stress (Fig. 5h). These results were verified using qRT-PCR of selected genes (Additional file 1: Fig. S3a–c). These data indicated that PepN treatment altered the mitochondrial functions of CD11c^high^ macrophages. Mitochondria from CD11c^high^ macrophages in the HDM group were also swollen and displayed signs of misshapen cristae while CD11c^high^ macrophages from the PepN + HDM group appeared unaltered (Fig. 5i). The change of mitochondrial morphology was also observed by confocal imaging (Fig. 5j). PepN pretreatment therefore protect the mitochondrial morphology of CD11c^high^ macrophages from HDM-induced damage.

We further examined mitochondrial health in these cell populations by measuring mitochondrial membrane potential (MMP), mitochondrial ROS (mt-ROS) and cellular oxygen consumption rate (OCR) of CD11c^high^ macrophages. A comparison with the HDM group indicated that PepN-pretreatment increased the MMP in CD11c^high^ macrophages (Fig. 5k, Additional file 1: Fig. S3d) and decreased mt-ROS production (Fig. 5j, l Additional file 1: Fig. S3e) and enhanced the OCR (Fig. 5m, Additional file 1: Fig. S3f). These results suggested that PepN-pretreatment enhances cellular mitochondrial respiratory function and alleviates dysfunction of CD11c^high^ macrophages caused by HDM. RNA-seq further revealed that PepN may modulate the activities of mitochondrial complexes although mitochondrial complex III and IV gene expression was similar and only complex I expression was altered for the two treatment groups (Additional file 1: Fig. S3g). We therefore examined the enzymatic activities of mitochondrial complex I and CD11c^high^ macrophages from PepN + HDM mice were more enzymatically active than were macrophages from the HDM group (Fig. 5n). Simultaneously, CD11c^high^ macrophages of PepN + HDM mice also had a more robust ATP production capacity (Fig. 5o). Together these data indicated that PepN-pretreatment prevented mitochondrial injury and promoted OXPHOS in CD11c^high^ macrophages.

Macrophage AMP-activated protein kinase (AMPK) activity can increase OXPHOS capacity by preventing the synthesis or secretion of proinflammatory cytokines from these cells [36, 37]. We therefore investigated whether enhanced oxidative phosphorylation following PepN treatment was related to AMPK pathway activity. We found that AMs from the BALF as well as lung homogenates of PepN + HDM mice displayed higher levels of AMPK phosphorylation and lower expression of subsequent signaling cascade proteins NF-κB and STAT3 than did the HDM mice (Fig. 5p, Additional file 1: Fig. S3h). In addition, PepN-pretreatment also induced M2-associated cytokine expression including Arg1 in the BALF AMs (Additional file 1:  Fig. S3i). In contrast, inflammatory cytokine expression of IL-1β in BALF AMs of PepN + HDM group was significantly decreased compared to the HDM group (Fig. 5q). After restimulation with HDM ex vivo, BALF AMs from PepN + HDM mice produced less IL-6 and TNF-α than did the HDM mice (Fig. 5r). Taken together, these results demonstrate that PepN-pretreatment enhances OXPHOS and p-AMPK expression in CD11c^high^ macrophages and this generated an overall anti-inflammatory phenotype.

### PepN treatment during macrophages maturation shapes their anti-inflammatory properties

**Fig. 6:**
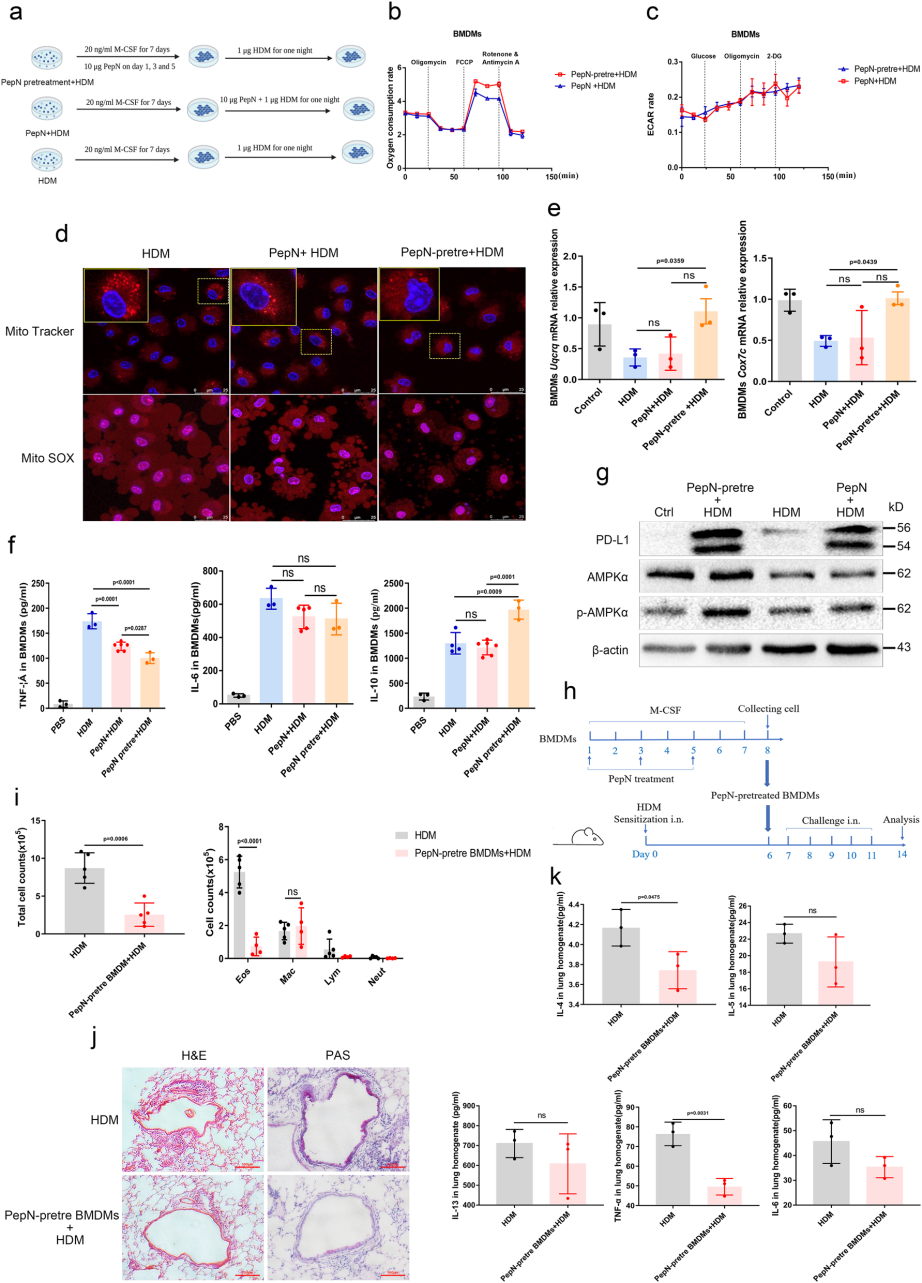
PepN-pretreatment shapes the anti-inflammatory property of macrophages during their maturation. **a** PepN (10 μg/mL) was supplied during and after the maturation of BMDM using HDM (1 μg/mL). **b**, **c** Oxygen consumption rate (**b**) and extracellular acidification rate (**c**) of BMDM (n = 3). **d** Mitochondrial morphology (Mito Tracker) and mt-ROS (Mito SOX) generation analysis of BMDM. Scale bars, 25 μm. **e** mRNA expression of mitochondrial electron transport-related genes *Uqcrq* and *Cox7c* in BMDM. **f** ELISA of cytokines in BMDM supernatants (n = 3–6). **g** p-AMPK and PD-L1 levels in BMDM. **h** Experimental design of PepN-pretreatment BMDM adoptive transfer asthma model in BALB/c mice. **i** Total inflammatory cell numbers and differential cell counts in BALF (n = 4–5). **j** Microscopic images of stained lung tissue sections of mice (200 × magnification). **k** ELISA of Th2 cytokines and TNF-α, IL-6 in lung homogenate (n = 4–5). Statistical analysis by one-way ANOVA with Tukey’s multiple-comparison test (**e**, **f**) or a two-tailed Student’s t test (**i, k**). Data representative images from 3 experiments (**f**). Data are represented as mean ± SD (**b**, **c**, **e**, **f**, **i** and **k**). ns, not significant

We further examined in vitro to verify whether PepN acts directly on macrophages (Fig. 6a, Additional file 1: Fig. S4a). HDM was used for macrophage maturation and the effects of PepN were then assessed prior to and following this treatment. PepN pretreatment followed by HDM increased the OCR compared with the cells that lacked the pretreatment (Fig. 6b), but the extracellular acidification rate (ECAR) did not differ between these two groups (Fig. 6c). The mitochondrial mass was also increased in the pretreatment group and displayed lower levels of mt-ROS generation (Fig. 6d). Identical experiments using THP-1-derived macrophages resulted in similar results except that the OCR was no difference (Additional file 1: Fig. S4b–d). The BMDM of PepN-pretreatment + HDM group also possessed increased levels of the electron transport chain-related genes *Uqcrq* and *Cox7c* (Fig. 6e) and exhibited reduced levels of TNF-α and increased IL-10 in tissue culture supernatants (Fig. 6f). The activation of AMPK was also increased with PepN pretreatment (Fig. 6g).

We further explored the effect of PepN-preprogramming by adoptively transferring BMDM from mice pretreated with PepN into HDM-sensitized recipient mice that were then challenged with a single dose of HDM (Fig. 6h). We found that BMDM pretreated with PepN induced protection against HDM-induced airway inflammation, as determined by reduced numbers of eosinophils in BALF (Fig. 6i), attenuated infiltration of inflammatory cells and mucus secretion in peribronchial and perivascular (Fig. 6j) and decreased levels of inflammatory cytokines IL-4 and TNF-α in lungs (Fig. 6k). These results demonstrated that PepN-pretreated BMDM acquired anti-inflammatory properties.

### PepN-elevated CD11c^high^ macrophages promote Tregs differentiation

**Fig. 7:**
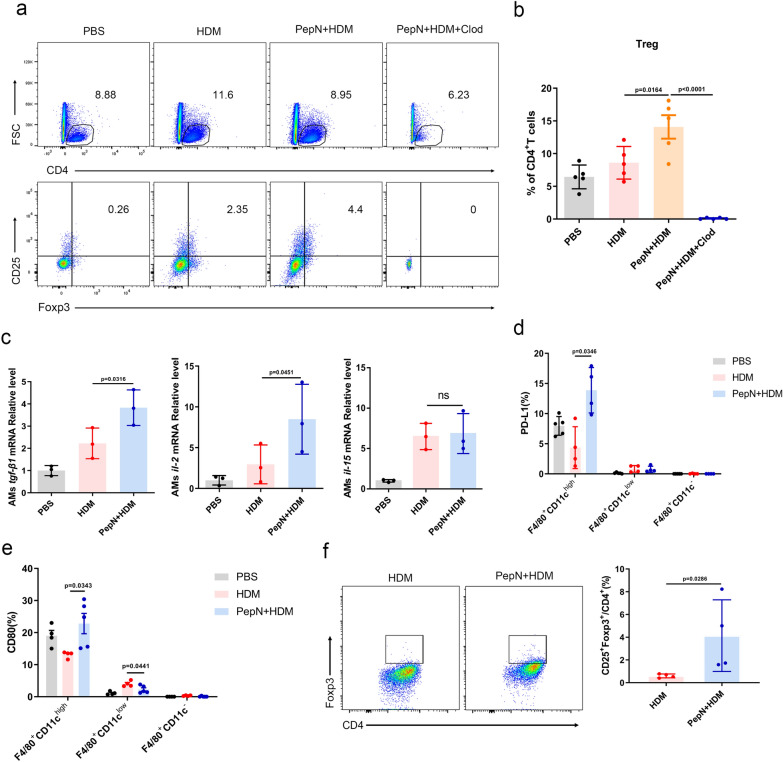
PepN-elevated CD11c^high^ macrophages promote Treg differentiation. **a**, **b** Flow cytometric analysis indicating proportions of Treg cells in lungs under the indicated conditions (n = 5–6). **c** mRNA relative levels of *Tgf-β*, *Il-2* and *Il-15* in CD11c^high^ macrophages. **d**, **e** Flow cytometric analysis of PD-L1 (**d**) and CD80 (**e**) expression in F4/80^+^CD11c^high/int/−^macrophages in lungs, respectively (n = 4–5). **f** Flow cytometric analysis of (%) Foxp3^+^Tregs in CD4^+^ T cells (n = 4). Statistical analysis by one-way ANOVA with Tukey’s multiple-comparison test (**b**–**e**), and a two-tailed student’s t test (**f**). Data are represented as mean ± SD (**b**–**f**). ns not significant

Tregs are an important immunosuppressive subset of CD4^+^T cells and are involved in regulating of immune balance. We employed chlodronate liposomes to deplete CD11c^high^ macrophages in PepN + HDM mice and found that depletion of these macrophages could inhibit Foxp3^+^Treg differentiation (Fig. 7a, b). Consistently, the expression of the Treg polarization related genes *Tgf-β1* and *Il-2* had increased in CD11c^high^ macrophages from PepN + HDM mice (Fig. 7c). These CD11c^high^ macrophages also displayed higher levels of the T cell costimulatory signals PD-L1 and CD80 than did HDM mice (Fig. 7d, e  Additional file 1: Fig. S5a). These results indicated that CD11c^high^ macrophages can regulate polarization of naïve CD4^+^T cells towards Treg cells. To further evaluate whether PepN-preprogrammed CD11c^high^ macrophages could directly elevate Tregs, CD11c^high^ macrophages were isolated from PepN + HDM mice and HDM mice and co-cultured respectively with naïve CD4^+^T cells for 3 days in the presence of α-CD3 and α-CD28 antibodies. The CD11c^high^ macrophages from PepN + HDM mice robustly promoted Treg differentiation when compared with those from HDM mice (Fig. 7f). These results indicated that PepN-preprogrammed macrophages can promote the differentiation of Tregs which further suppress airway inflammation.

## Discussion and conclusions

**Fig. 8:**
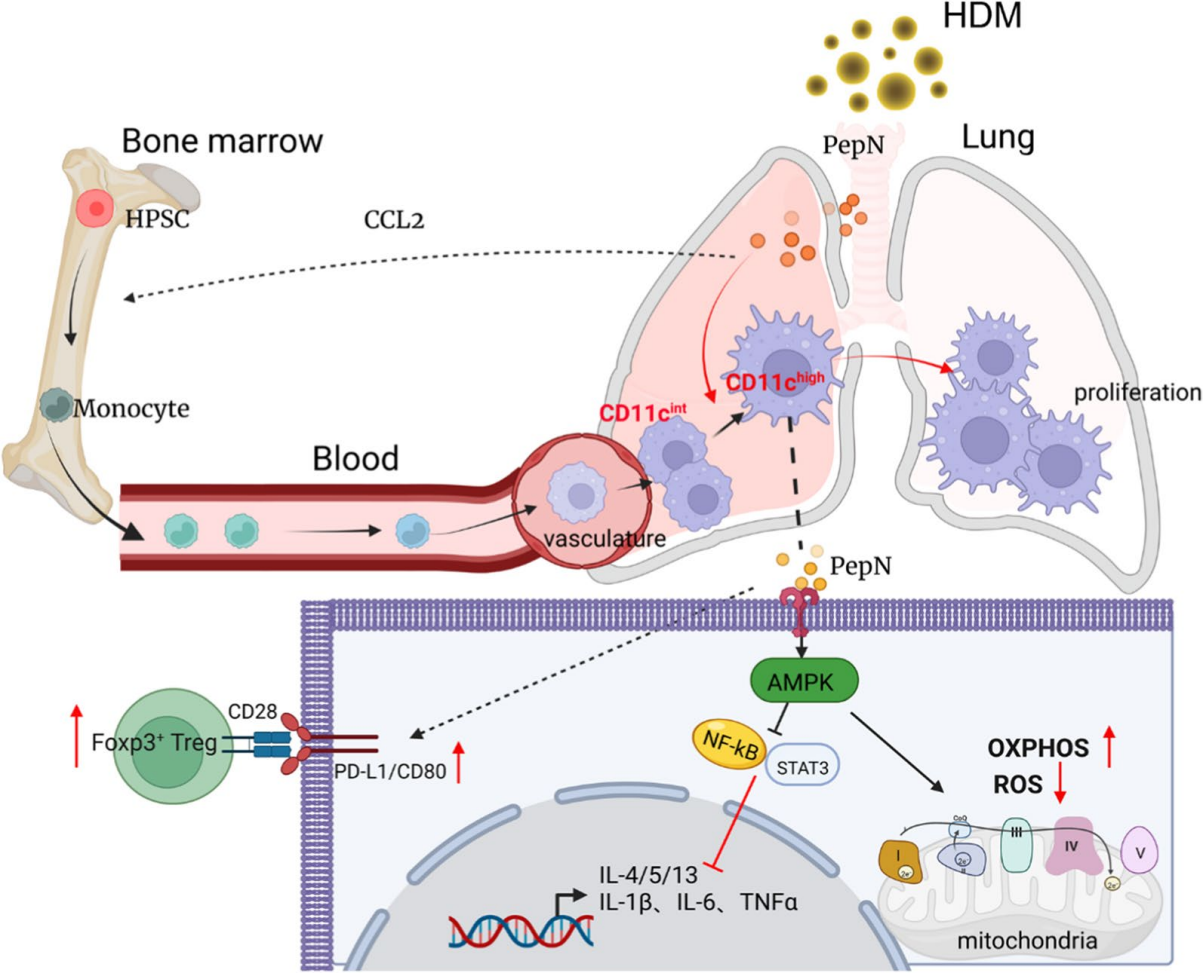
Proposed mechanism of PepN on CD11c^high^ macrophages-dependent anti-inflammatory effects in allergic asthma. In asthmatic mice, PepN promotes the proliferation of lung-resident CD11c^high^ macrophages in situ and stimulates the recruitment of BMDM to the respiratory tract and differentiation into CD11c^int^ macrophage cell pool. Evanescence of CD11c^int^ macrophages in PepN-treated mice was found to be phenotypical transformation into pulmonary CD11c^high^ macrophages. Furthermore, PepN-preprogramed CD11c^high^ macrophages acquired an anti-inflammatory property by shaping the metabolic preference of OXPHOS and upregulated the expression of costimulatory molecules to promote Treg differentiation, thus exerting a protective effect against asthmatic airway inflammation. Overall, our data suggest that the microbial component PepN is a potential prevention and treatment strategy for allergic asthma by targeting CD11c^high^ macrophages and this provides new theoretical foundation for the hygiene hypothesis

In this study, we revealed that PepN-pretreatment through the respiratory tract inhibited HDM-induced airway inflammation in asthmatic mice and exerted anti-inflammatory effects by altering the number and phenotypes of lung macrophages. We also found that PepN recruited BMDM and phenotypically transformed them into pulmonary CD11c^high^ macrophages. Simultaneously, PepN-preprogramed CD11c^high^ macrophages acquired an anti-inflammatory property by shaping the metabolic preference of OXPHOS and upregulated the expression of costimulatory molecules to promote Treg differentiation, thus exerting a protective effect against asthmatic airway inflammation (Fig. 8).

Resident macrophages are maintained by self-renewal and circulating monocytes do not contribute to these populations in tissue homeostasis [38]. Thus, proliferation is the major source of AMs for self-renewal in situ during infection and this has been demonstrated for a broad range of pathogens [39–41]. However, BMDM can enter the alveolar space and contribute to the macrophage pools in lung tissues and these show similar gene expression profiles as macrophages derived from the yolk sac [42]. Moreover, changes in the lung microenvironment can modulate BMDM recruitment, which has been demonstrated in MuHV-4 infections that provide protection against airway allergy [4]. Consistent with these previous studies, we found that PepN-pretreatment could promote the proliferation of CD11c^high/int/−^ macrophages and also could inhibit their apoptosis. Moreover, PepN-pretreatment could recruit BMDM to lung tissues that was demonstrated by CCL2 expression and the chimeric mice model. Nevertheless, we found that the number of CD11c^int^ macrophages in lungs was reduced in PepN-pretreatment HDM-induced asthmatic mice.

To explain this phenomenon, mice were treated with PepN alone and then the populations of CD11c^high/int/−^ macrophages in lungs were examined at different time points. CD11c^int^ macrophages were converted to CD11c^high^ macrophages. The mouse chimera revealed that PepN could recruit myelomonocytic-derived CD11c^int^ macrophages to lung tissues that were then transformed into CD11c^high^ macrophages. In order to demonstrate whether the phenotypic transformation of CD11c^int^ macrophages induced directly by PepN treatment, CD11c^int^ macrophages separated via FCM were treated with PepN alone in vitro, but no phenotypic transformation was observed in macrophages (data not shown). We presume that the regulation of macrophages by PepN in vivo may be a complex process involving interaction between multiple cytokines or immune cells. In summary, this study indicated that PepN-pretreatment increased CD11c^high^ macrophage numbers by inhibiting their apoptosis and promoting proliferation while recruiting and converting BMDM to the CD11c^high^ phenotype.

Previous studies on AMs derived from naïve mice indicated these cells are central in suppressing immune responses to inhaled antigens deposited on the alveolar surface [43], and AMs express high level of CD11c [44]. We found that CD11c^high^ macrophages were increased both in PepN + HDM and HDM groups compared with PBS group while they functionally differed. We use depletion and adoptive transfer experiments to confirm that CD11c^high^ macrophages from PepN + HDM mice have anti-inflammatory properties, while CD11c^high^ macrophages from HDM mice have pro-inflammatory properties consistent with previous reports [45, 46]. Our transcriptome analysis also supported these conclusions.

Macrophage activation is accompanied by strictly regulated intracellular metabolic reprogramming and associated with cell proliferation, differentiation and functional activates due to the maintenance of immune homeostasis and in response to acute inflammatory conditions [25]. Metabolic reprogramming usually occur during differentiation of monocyte to macrophage [47], to meet their demand for energy and biosynthesis, and also to provide critical metabolic end products for macrophage effector functions [48, 49]. Inhibition of fatty acid synthesis, glycolysis or boosting FAO or OXPHOS play important roles in these processes and imbues macrophages with anti-inflammatory properties [29]. Our results demonstrated that CD11c^high^ macrophages from PepN-pretreated mice exhibited higher OXPHOS than did the HDM mice and included up-regulated expression of genes involved in the mitochondrial respiratory chain complex assembly and function specifically for complex I. In addition, mt-ROS were inhibited and MMP was enhanced. Simultaneously, PepN pretreatment could protect mitochondrial morphology of CD11c^high^ macrophages from HDM-induced damage. Moreover, both in vivo and in vitro experiments indicated that PepN pretreatment could inhibit the expression of pro-inflammatory TNF-α and IL-1β and promote the expression of the anti-inflammatory IL-10. Therefore, our results suggest that CD11c^high^ macrophages of PepN pretreated mice exhibit higher OXPHOS and display an anti-inflammatory effect.

Tregs are important immunosuppressive cells and play important protective roles in allergic asthma by inhibiting Th2 response [50]. TR-AMs exposure to allergens suppresses their ability to generate Treg cells coincident with blocking airway tolerance [51]. However, our results demonstrated that the depletion of CD11c^high^ macrophages in PepN + HDM mice by chlodronate liposomes inhibited the differentiation of Foxp3^+^Tregs. Expression of Treg polarization-related genes *Tgf-β1* and *Il-2* and T cell costimulatory molecules PD-L1 and CD80 increased in CD11c^high^ macrophages of PepN + HDM mice. The experiments of CD11c^high^ macrophages co-cultured with naive CD4^+^T cells also confirmed that CD11c^high^ macrophages from PepN + HDM mice were potent in promoting Treg differentiation. These results indicated that PepN pretreatment can induce Treg polarization and this further inhibited airway inflammation in asthmatic mice.

Regulation between immune cells is very complex, more research and trials are needed to provide a theoretical basis for the hygiene hypothesis. Here, we disclosed a novel microbial component with immunomodulatory properties PepN can program macrophages during maturation to anti-inflammatory effects by shaping the metabolic preference for OXPHOS. However, there are still limitations in our study: The evidence of phenotypic transformation of BMDM preprogramed by PepN in lungs remains insufficient. Moreover, the effects of CD11c^int^ macrophages and CD11c^−^ macrophages after PepN-pretreated have not been validated. Nonetheless, this study adds evidence to the anti-inflammatory effects of the *S. pneumoniae* PepN in allergic asthma and provides a partial explanation for its mechanism of action. Our study provides a new idea for prevention and treatment of allergic asthma by targeting CD11c^high^ macrophages and new theoretical foundation for the hygiene hypothesis.

### Supplementary Information

**Additional file 1: Table S1. **Primers used in quantitative PCR. **Fig. S1.** Effect of PepN-pretreatment on macrophages in lungs of asthmatic mice. **Fig. S2.** Effects of PepN-treatment on the proliferation and transformation of CD11chigh/int macrophages in lungs. **Fig. S3.** PepN-pretreatment changes the cell metabolism and phenotypes of CD11chigh macrophages. **Fig. S4.** PepN-pretreatment influences the anti-inflammatory property of THP-1-derived macrophages. **Fig. S5.** PepN-pretreatment increases the expression of PD-L1 in CD11chigh macrophages.

## Data Availability

The datasets used and/or analyzed during the current study are available from the corresponding author on reasonable request.

## Abbreviations

AM: Alveolar macrophage
AMPK: AMP-activated protein kinase
BMDM: Bone marrow derived macrophage
DGEs: Differentially expressed genes
FAO: Fatty acid oxidation
GSEA: Gene set enrichment analysis
HDM: House dust mite
IM: Interstitial macrophage
MMP: Mitochondrial membrane potential
Mo-AM: Monocyte-derived alveolar macrophage
mt-ROS: Mitochondrial ROS
OCR: Oxygen consumption rate
OVA: Ovalbumin
OXPHOS: Oxidation phosphorylation
PepN: *Streptococcus pneumoniae* Aminopeptidase N
ROS: Reactive oxygen species
TR-AM: Tissue-resident alveolar macrophage
