# Supplementary material for: The preprogrammed anti-inflammatory phenotypes of CD11chigh macrophages by Streptococcus pneumoniae aminopeptidase N safeguard from allergic asthma
Source: J Transl Med. 2023 Dec 11;21:898. doi: 10.1186/s12967-023-04768-2 (PMC10712085; doi:10.1186/s12967-023-04768-2)
Supplement: Supplementary file 1 — Additional file 1: Table S1. Primers used in quantitative PCR. Fig. S1. Effect of PepN-pretreatment on macrophages in lungs of asthmatic mice. Fig. S2. Effects of PepN-treatment on the proliferation and transformation of CD11chigh/int macrophages in lungs. Fig. S3. PepN-pretreatment changes the cell metabolism and phenotypes of CD11chigh macrophages. Fig. S4. PepN-pretreatment influences the anti-inflammatory property of THP-1-derived macrophages. Fig. S5. PepN-pretreatment increases the expression of PD-L1 in CD11chigh macrophages. [file 12967_2023_4768_MOESM1_ESM.docx]

Additional file 1

**The preprogrammed anti-inflammatory phenotypes of CD11c^high^ macrophages by *Streptococcus pneumoniae* aminopeptidase N safeguard from allergic asthma**

Shifei Yao, Danlin Weng, Yan Wang, Yanyu Zhang, Qi Huang, Kaifeng Wu, Honghui Li, Xuemei Zhang, Yibing Yin, Wenchun Xu

This file includes Supplemental Table 1 and Figures 1-5.

**Table S1. Primers used** **in quantitative PCR.**

| Gene name | Forward primer | Reverse primer |
| --- | --- | --- |
| *Il-4* | GGTCTCAACCCCCAGCTAGT | GCCGATGATCTCTCTCAAGTGAT |
| *Il-5* | TCAGGGGCTAGACATACTGAAG | CCAAGGAACTCTTGCAGGTAAT |
| *Il-13* | TGAGCAACATCACACAAGACC | GGCCTTGCGGTTACAGAGG |
| *Uqcc2* | GAGCTTAGCACGACTGCATTC | TGGTGGGCGCAAACTTCTC |
| *Mrpl52* | CACCCCCAATGAAAGGCCAA | CTGAGCTTCCATGCCTGTATTC |
| *Uqcrq* | CCTACAGCTTGTCGCCCTTT | GATCAGGTAGACCACTACAAACG |
| *Cox7c* | ATGTTGGGCCAGAGTATCCG | ACCCAGATCCAAAGTACACGG |
| *Gpx1* | CCACCGTGTATGCCTTCTCC | AGAGAGACGCGACATTCTCAAT |
| *Gpx4* | TGTGCATCCCGCGATGATT | CCCTGTACTTATCCAGGCAGA |
| *Il-2* | TGAGCAGGATGGAGAATTACAGG | GTCCAAGTTCATCTTCTAGGCAC |
| *Il-15* | CATCCATCTCGTGCTACTTGTG | GCCTCTGTTTTAGGGAGACCT |
| *Tgf-β1* | CTTCAATACGTCAGACATTCGGG | GTAACGCCAGGAATTGTTGCTA |
| *S100a9* | GCACAGTTGGCAACCTTTATG | TGATTGTCCTGGTTTGTGTCC |
| *Ndufa1* | ATGTGGTTCGAGATTCTCCCT | TTTGTGGATGTACGCAGTGGA |
| *Ndufb7* | CGGCGCTATCTGTGGGATG | CTGTCTCGCTTGCACTTCAG |
| *Ndufa4* | TCCCAGCTTGATTCCTCTCTT | GGGTTGTTCTTTCTGTCCCAG |
| *Cox4i1* | ATTGGCAAGAGAGCCATTTCTAC | CACGCCGATCAGCGTAAGT |
| *Uqcr10* | ATCCCTTCGCGCCTGTACT | GTGCTCGTAGATCGCGTCT |
| *Uqcrb* | GGCCGATCTGCTGTTTCAG | CATCTCGCATTAACCCCAGTT |
| *Tnf-α* | CTGAACTTCGGGGTGATCGG | GGCTTGTCACTCGAATTTTGAGA |
| *Cd86* | CTGGACTCTACGACTTCACAATG | AGTTGGCGATCACTGACAGTT |
| *iNOS* | GTTCTCAGCCCAACAATACAAGA | GTGGACGGGTCGATGTCAC |
| *Arg1* | TGTCCCTAATGACAGCTCCTT | GCATCCACCCAAATGACACAT |
| *Cd206* | GAGGGAAGCGAGAGATTATGGA | GCCTGATGCCAGGTTAAAGCA |
| *Il-10* | CTTACTGACTGGCATGAGGATCA | GCAGCTCTAGGAGCATGTGG |
| *β-actin* | GTGACGTTGACATCCGTAAAGA | GCCGGACTCATCGTACTCC |

Figure S1


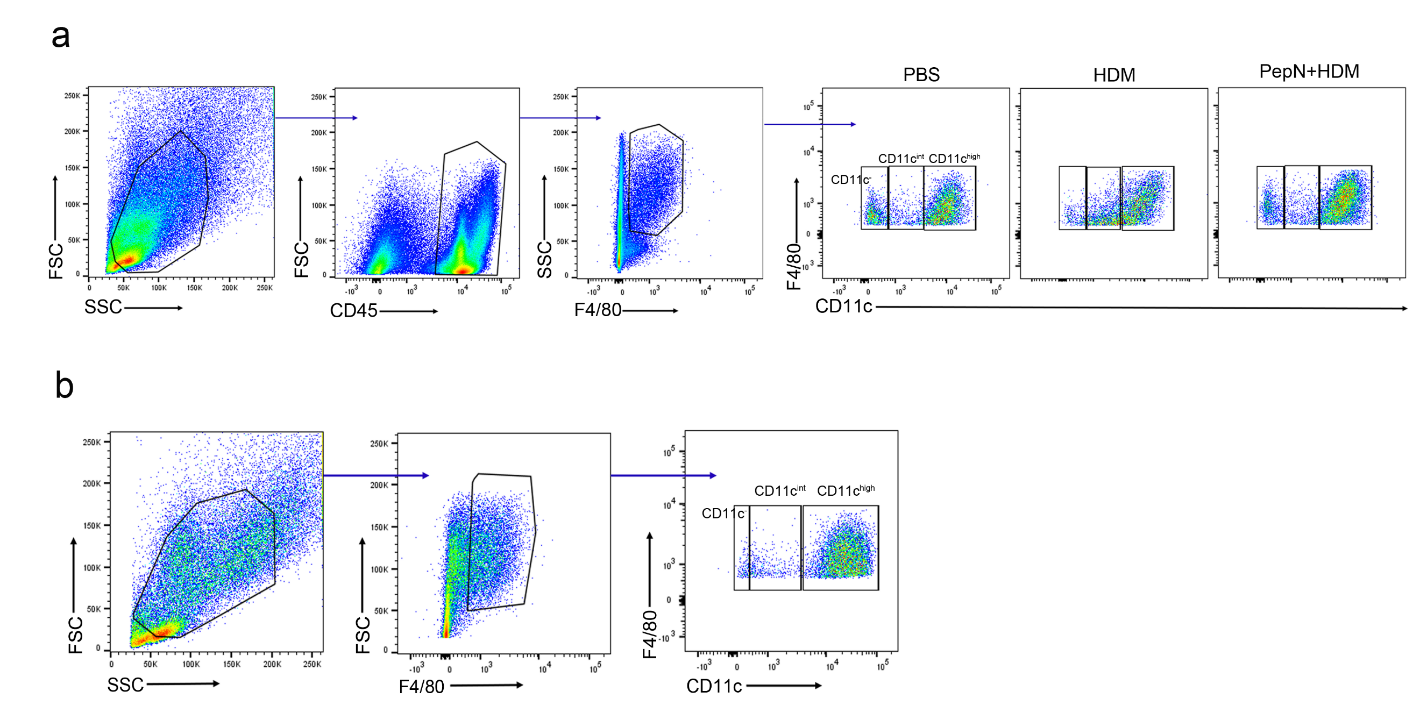


**Fig. S1** Effect of PepN-pretreatment on macrophages in lungs of asthmatic mice. **a** Gating strategy identifying CD11c^high/int/-^ macrophages in the lung (developed in Fig. 1**h, i**) applied at day 14 to PBS, HDM and PepN+HDM group mice. **b** Gating strategy identifying CD11c^high/int/-^ macrophages in BALF.

Figure S2


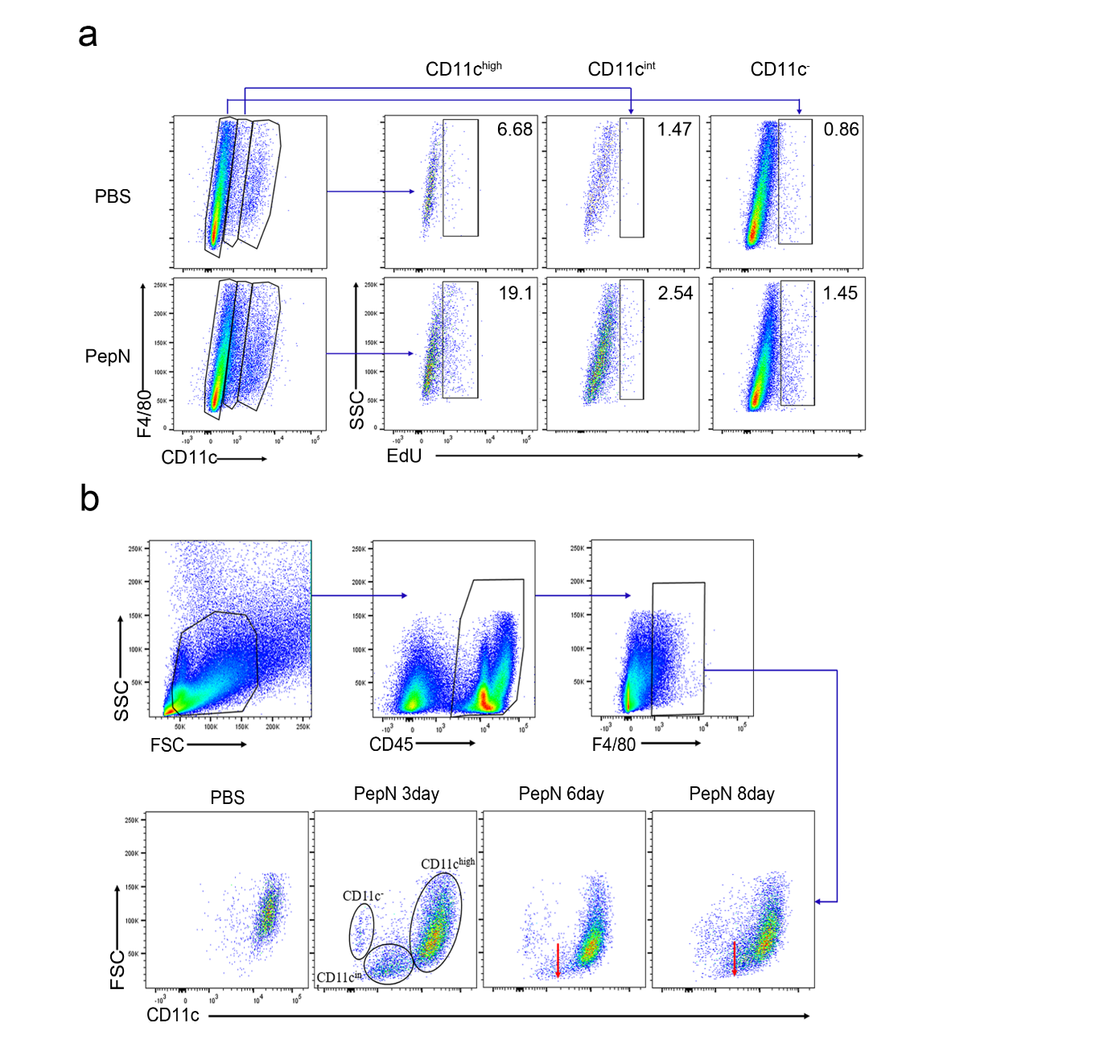


**Fig. S2** Effects of PepN-treatment on the proliferation and transformation of CD11c^high/int^ macrophages in lungs. **a** Dot plots showing percentage of EdU^+^ cells in CD11c^high/int/-^ macrophages in lungs after i.n. PepN treatment alone. **b** Gating strategy identifying the change of CD11c^high^ and CD11c^int^ macrophages phenotypes in lungs after i.n. PepN treatment alone.

Figure S3


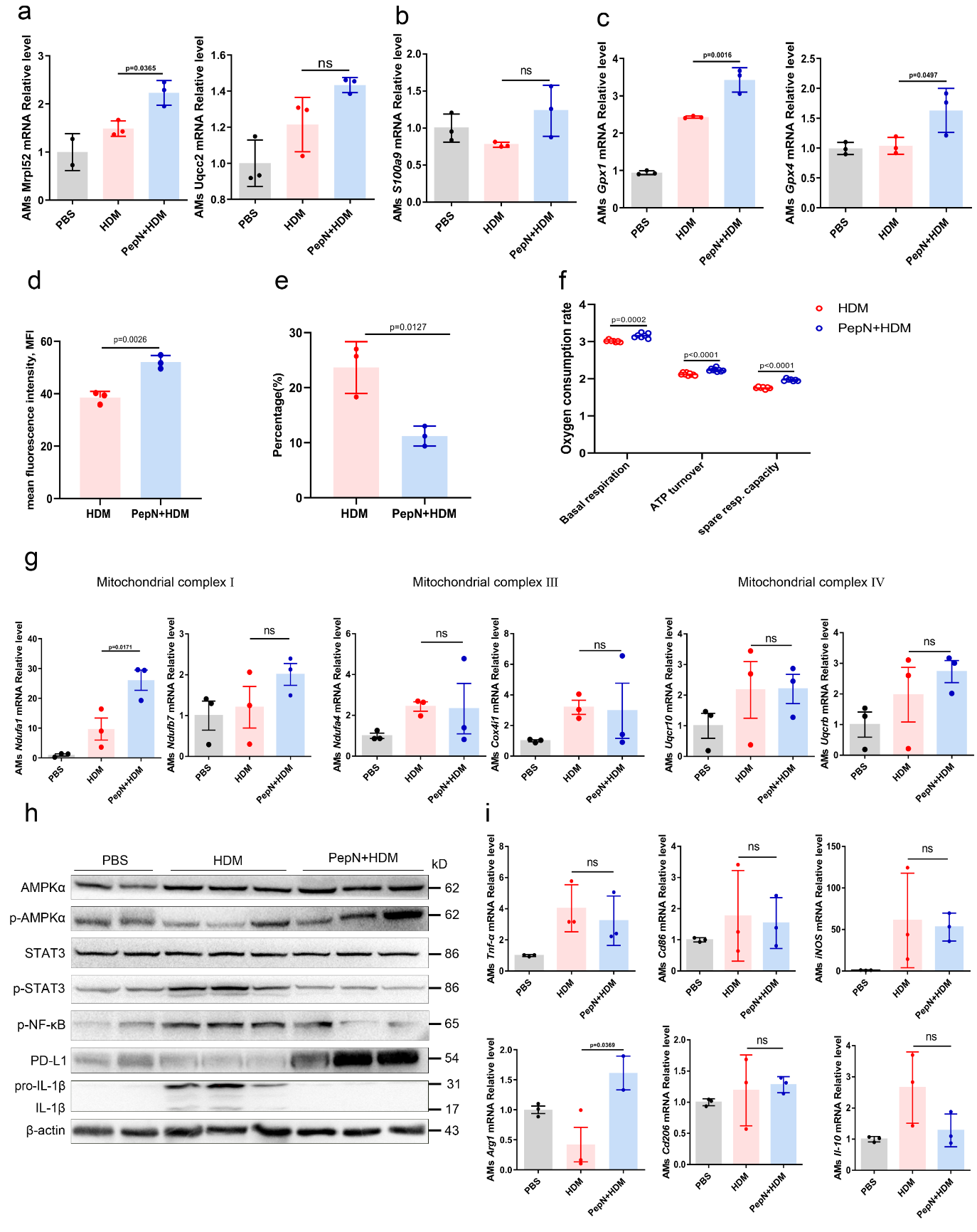


**Fig. S3** PepN-pretreatment changes the cell metabolism and phenotypes of CD11c^high^ macrophages. **a-c** mRNA relative level of mitochondrial translation-related genes *Uqcc2* and *Mrpl52*, antioxidase-related genes *Gpx1* and *Gpx4*, and wound healing-related gene *S100a9* of CD11c^high^ macrophages, respectively (n = 3). **d, e** Mitochondrial membrane potential (d) and mt-ROS (e) of CD11c^high^ macrophages were detected by flow cytometry (n = 3). **f** Oxygen consumption rate (OCR) of CD11c^high^ macrophages with indicated treatments. **g** mRNA relative level of mitochondrial complex I-related gene *Ndufa1* and *Ndufb7*, mitochondrial complex III-related gene *Ndufa4* and *Cox4i1*, and mitochondrial complex IV-related gene *Uqcr10* and *Uqcrb* of CD11c^high^ macrophages, respectively (n = 3). **h** Activation of AMPK, STAT3 and NF-κB signaling pathway in lung tissues were detected by western blot. (n = 2-3). **i** mRNA expression of M1/M2 polarization markers of CD11c^high^ macrophages (n = 3). Statistical analysis by a two-tailed Student’s t test (**d-f**) or one-way ANOVA with Tukey’s multiple-comparison test (**a-c, g** and **i**). Data representative images from 3 experiments (**h**). Data are represented as mean ± SD (**a-g** and **i**). ns, not significant.

Figure S4


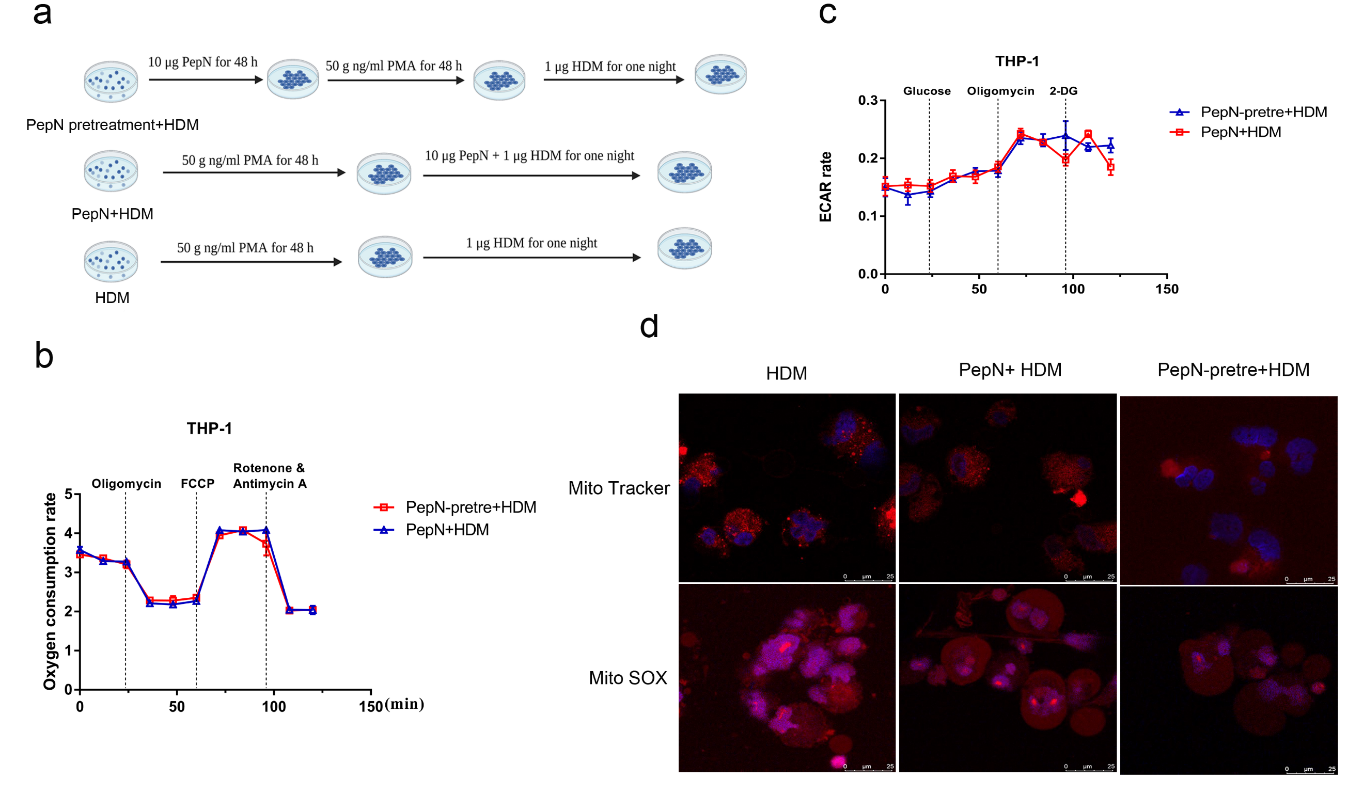


**Fig. S4** PepN-pretreatment influences the anti-inflammatory property of THP-1-derived macrophages. **a** PepN (10 μg/mL) was supplied before the maturation of THP1-derived macrophages, the HDM (1 μg/mL) responses of matured macrophages were examined. **b, c** Oxygen consumption rate (OCR) and extracellular acidification rate (ECAR) of THP1-derived macrophages were detected (n = 3). **d** Influence of PepN pre-treatment on the mitochondrial morphology (Mito Tracker) and the generation of mt-ROS (Mito SOX) in THP1-derived macrophages. Scale bars, 25 μm. Data are represented as mean ± SD (**b, c**).

Figure S5


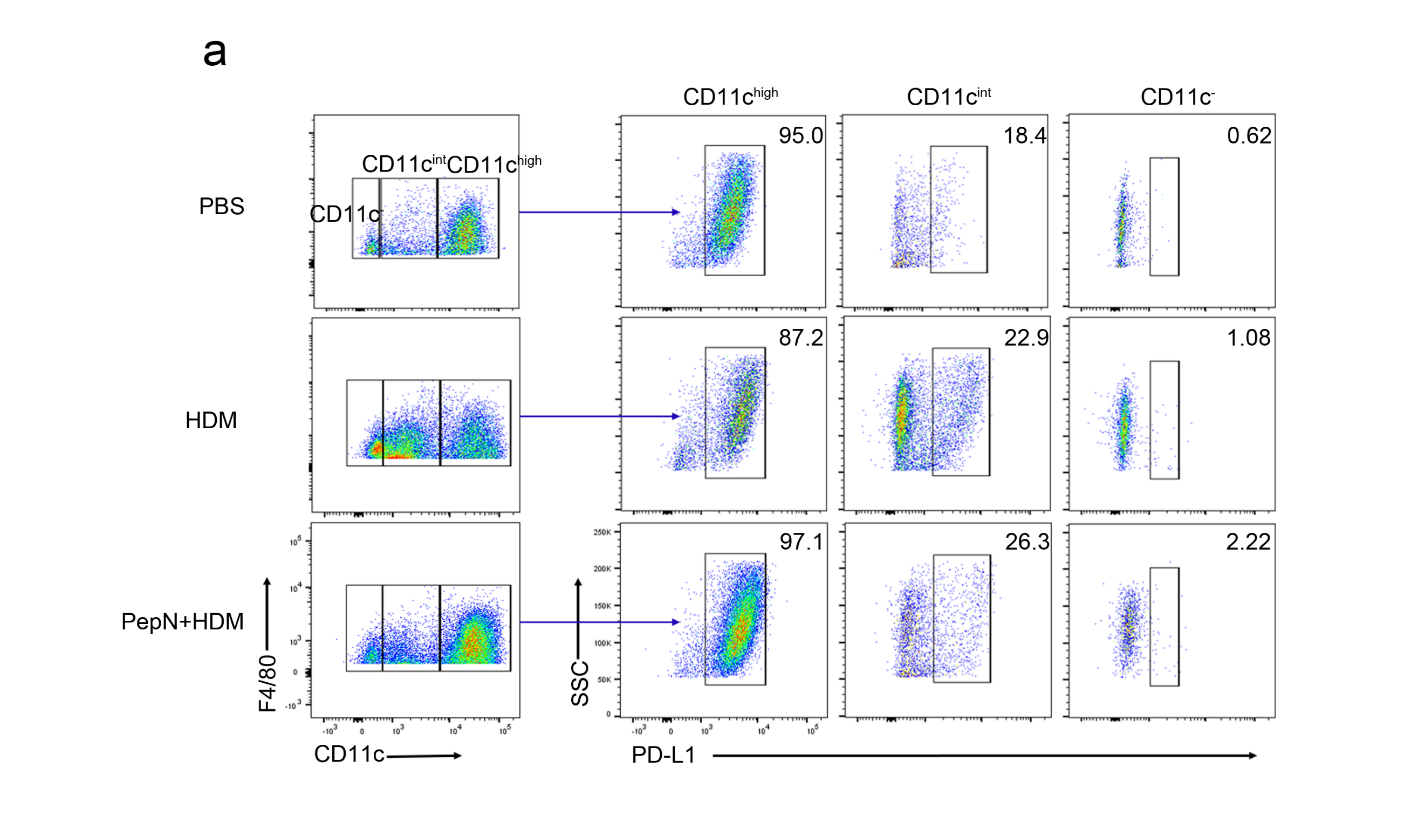


**Fig. S5** PepN-pretreatment increases the expression of PD-L1 in CD11c^high^ macrophages. **a** Dot plots showing percentage of PD-L1^+^ cells in CD11c^high/int/-^ macrophages from PBS, HDM and PepN+HDM group mice.
